# Sulfonic acid-functionalized polyallylamine (sevelamer) as an efficient reusable strong solid acid catalyst for the synthesis of xanthenes derivatives

**DOI:** 10.1186/s13065-019-0609-4

**Published:** 2019-07-26

**Authors:** Xian-Liang Zhao, Makombe Shelton, Ke-Fang Yang

**Affiliations:** 1 School of Biological and Chemical Engineering, Zhejiang University of Science and Technology, Hangzhou, 310023 People’s Republic of China; 2 Key Laboratory of Organosilicon Chemistry and Material Technology of Ministry of Education, Hangzhou Normal University, Hangzhou, 310012 China

**Keywords:** Sevelamer, Sulfonic acid, 1,8-Dioxo-octahydroxanthene, Recycle, Water

## Abstract

Sevelamer (polyallyamine resin)-supported sulfonic acid (S-SO_3_H) has been prepared from the reaction of sevelamer with chlorosulfonic acid and characterized using FT-IR spectroscopy, scanning electronmicroscopy (SEM) and thermogravimetric analysis (TGA). The catalytic activity of S-SO_3_H was investigated in the synthesis of 1,8-dioxo-octahydroxanthene derivatives. All of the reactions were fast and gave excellent yields. The catalyst was easily recovered and reused for 5 runs without significant loss of its catalytic activity.
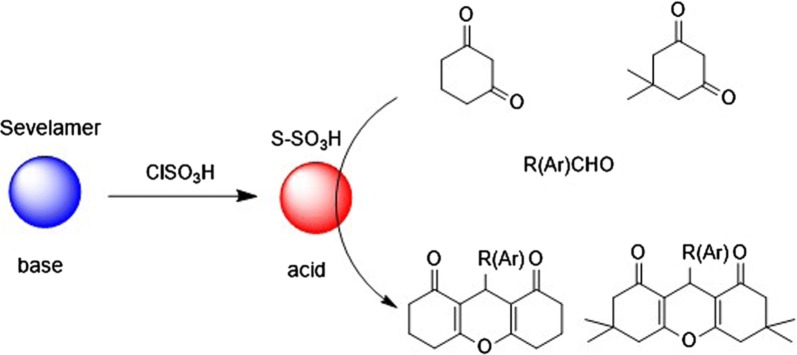

## Introduction

Heterogeneous catalysis involving functional polymers has been very important over the past few decades [1–3]. Catalysts immobilized on a functional polymer support shows efficiency strategy for the isolation and catalyst recycling, leading to economic and environmental advantages [4, 5].

The most commonly used polymer-supports involves polystyrene cross-linked with divinylbenzene (PS/DVB) [6–8]. However, there are some drawbacks to PS/DVB. The number of chemical modification reactions, such as chloromethylation, sulfonation that can be carried out to introduce functional groups onto the polystyrene (PS)-based beads are limited and the reactions themselves may be difficult to control. On the other hand, the induced functional groups are limited and reduce the number of catalytic sites. It’s useful that functional polymers contain reactive groups that can be used directly for the further introduction of functional groups. The amount of induced functional groups can be easily controlled. Sevelamer is a co-polymer of epichlorohydrin and polyallylamine [9]. Sevelamer is an environmentally friendly solid material with excellent mechanical strength and stability. This compound contains abundant amine groups, which can be easily transformed into various functionality.

The use of solid acids in organic synthesis and the industrial manufacture of materials is of increasing importance [10]. Heterogeneous acid catalysts such as solid acid zeoilte, solid superacid sulfated zirconia and Nafion have been explored in both organic synthesis and large scale industry. Polymer supported acids, such as polystyrene resin and perfluorinated ion exchange polymers, have also been prepared and widely used in organic synthesis [11–13]. These catalysts are suitable for a variety of synthetic conditions. However, they are associated with one or more disadvantages such as lower reactivity, the use large quantities of the solid support and not being suitable for reactions conducted in aqueous media.

In the past decade, xanthene and its derivatives has been widely used in pharmaceuticals, drug discovery, dyes, fluorescent materials and biochemistry. Xanthenes, especially 1,8-dioxo-octahydroxanthenes, are prepared using a range of different methods, due to their wide range of synthetic, industrial and pharmacological applications [14–18]. Various catalysts have been used for the preparation of these xanthenes. A wide range of catalysts such as MCM-41-SO_3_H [19], SbCl_3_/SiO_2_ [20], *p*-dodecylbenzene sulfonic acid [21], triethylbenzylammonium chloride [22], β-cyclodextrin SO_3_H [23], Amberlyst-15 [24], diammonium hydrogen phosphate [25] and nano-TiO_2_ [26] have been used for the preparation of 1,8-dioxo-octahydroxanthenes. On the other hand, there have been several reports describing the use of SO_3_H immobilized on polymers towards the synthesis of 1,8-dioxo-octahydroxanthenes. However, many of these methods suffer from disadvantages such as low yields, long reaction times, the use of harmful volatile organic solvents and tedious work-ups.

Recently, we have focused our attention on the use of polymeric catalysts as recyclable catalysts in organic synthesis in environmentally sustainable chemistry [27]. Functional polymers used as the solid support are very important for the definition of efficient synthetic procedures especially when safer reaction media, ecofriendly alternatives to organic solvents or solvent-free conditions (SolFC) are used.

## Experimental

### Instruments

TGA was carried out on a Mettler-Toledo 851E instrument. ^1^H NMR spectra were recorded on a 400 MHz spectrometer (Varian, Palo Alto, USA) in DMSO solution. Chemical shift values are given in parts per million. IR spectra were recorded on a Perkin Elmer (model: spectrum BX) FT-IR Spectrometer using KBr pellets.

### Preparation of polyallylamine hydrochloride

Allylamine (15.0 g) was slowly added to concentrated HCl (26.8 g) maintaining the temperature below 10 °C. Nitrogen gas was introduced into the flask after 30 min and the solution was heated at 50 °C. Afterwards, 2,2′-diamidinyl-2,2′-azo-propane dihydrochloride (1.0 g) was added to the solution. The reaction mixture was stirred at 50 °C for 24 h. A second portion of 2,2′-diamidinyl-2,2′-azo-propane dihydrochloride (1.0 g) was then added to the solution. The reaction mixture was maintained for another 24 h at 50 °C. The pale yellow, transparent and viscous solution obtained in this process was added into a large amount of methanol and a white polymer precipitate was subsequently produced. This precipitate was filtered and extracted with methanol using a Soxhlet extractor for 10 h. After being dried at 50 °C, polyallylamine hydrochloride was obtained (15.6 g).

### Typical procedure for the preparation of support s

Polyallylamine hydrochloride (5.0 g) was dissolved in water (75.0 mL) to obtain a clear solution at 30 °C. The solution was further cooled to 10 °C. Then, NaOH (1.38 g) was added to the solution at 10 °C and stirred for 30 min. Then, 40 mL of toluene and SPAN-85 (0.2 g) were added to the mixture at 10 °C. The reaction mixture was maintained for another 30 min at 10 °C. The temperature was then increased to 55 °C and maintained at this temperature for 15 min. Afterwards, epichlorohydrin (0.45 g) was added to the reaction mixture at 55 °C and maintained at this temperature for 3 h. The reaction mixture was cooled to room temperature. Sevelamer hydrochloride was then filtered, washed with water and dried in vacuo. The compound was then soaked in NaOH solution (10.0 g, 10% w/v) at room temperature for 24 h. The product was filtered and washed with hot water until it became pH neutral. Finally, the product was dried at 60 °C for 12 h under vacuum. **S** (3.22 g, 94%) was obtained as a pale-yellow solid.

### Preparation of S-SO_3_H

A suspension of sevelamer (4.0 g) and Na_2_CO_3_ (7.35 g) in dry CH_2_Cl_2_ (20 mL) was added to a suction flask. Then, chlorosulfonic acid (3.0 mL, 30 mmol) was added dropwise over a period of 15 min at room temperature while the mixture was stirred slowly at room temperature. After the addition was complete, the mixture was stirred for 12 h. Then, the CH_2_Cl_2_ was removed by filtration under reduced pressure and the solid powder dissolved in water (200.0 mL). The mixture was filtered again. The solid powder was washed with water, then soaked in H_2_SO_4_ solution (10.0 g, 10% v/v) at room temperature for 24 h. The product was filtered and washed with water until it became pH neutral. Finally, the product was dried at 70 °C for 12 h under vacuum. A yellow solid of polyallylamine-supported sulfonic acid was obtained (6.1 g).

## Results and discussion

### Preparation of S-SO_3_H

**Scheme 1:**
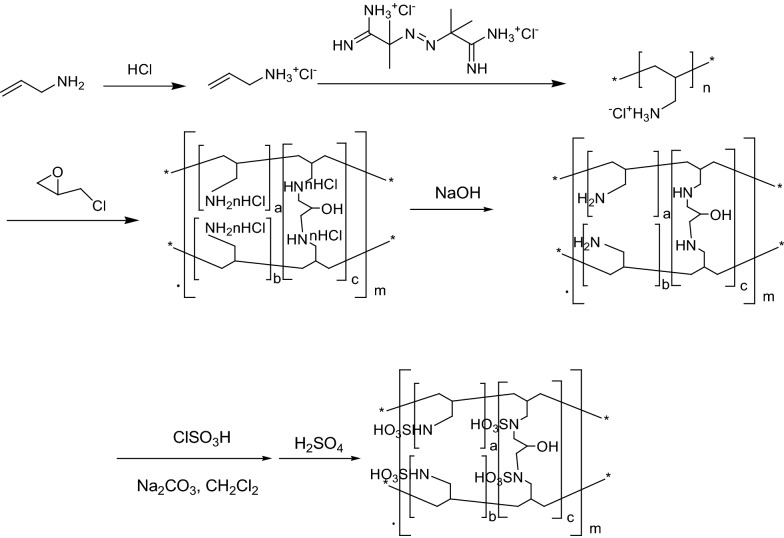
Synthesis of S-SO_3_H

The synthetic strategy used to prepare S-SO_3_H is shown in Scheme 1. At first, sevelamer was synthesized according to our previous work. Then, sevelamer was used to support SO_3_H.

### Catalyst characterization

#### Scanning electron microscopy

**Fig. 1:**
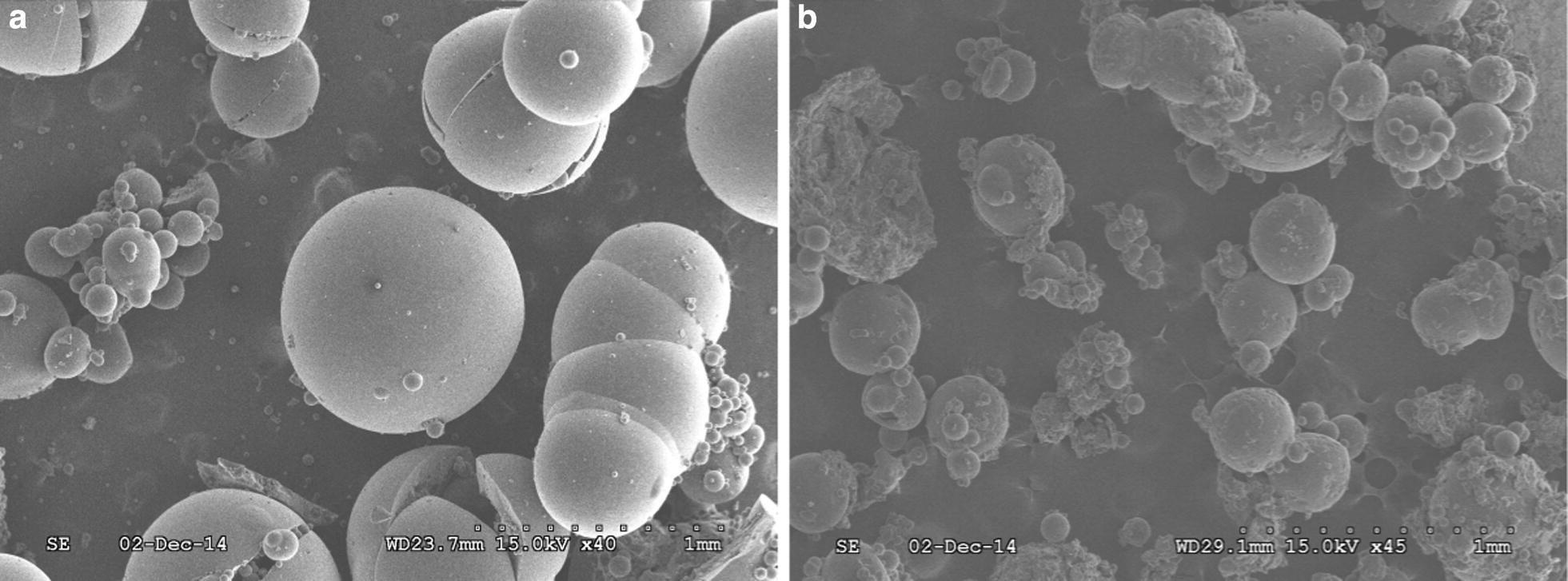
SEM image of resin sevelamer (**a**) and resin S-SO_3_H (**b**). **a** sevelamer before supported by ClSO_3_H, **b** sevelamer after supported by ClSO_3_H

Scanning electron micrographs which shows the preparation of sevelamer support are reported in Fig. 1. Resin sevelamer (Fig. 1a) shows a fine and uniform surface texture as compared to (Fig. 1b). Resin S-SO_3_H (Fig. 1b) show rough contamination with fragments on its surface.

#### FT-IR spectroscopy

**Fig. 2:**
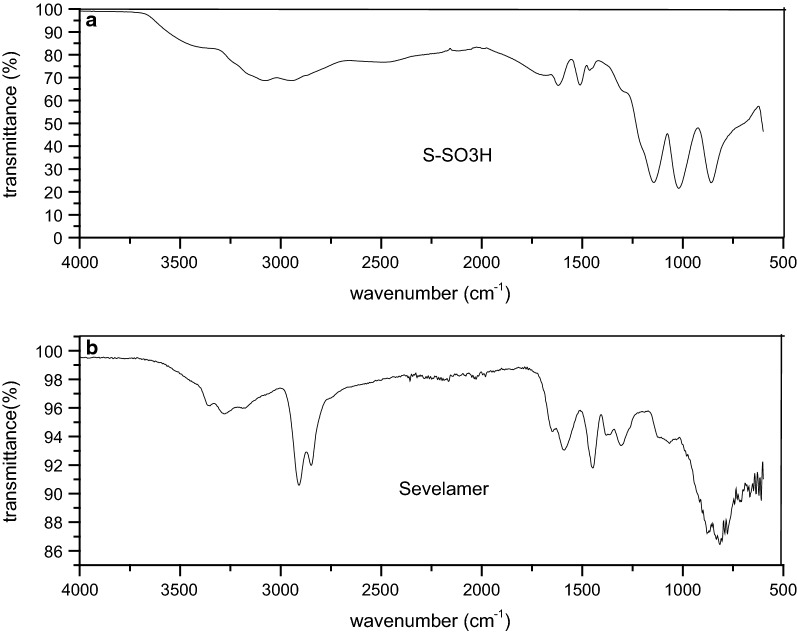
FTIR spectra of resin sevelamer (**b**) and resin S-SO_3_H (**a**). **a** sevelamer after supported by ClSO_3_H, **b** sevelamer before supported by ClSO_3_H

The FT-IR spectra of sevelamer and S-SO_3_H are shown in Fig. 2. The absorbance bands ranging between 3200–3500 cm^−1^ were shows acid adsorption (Fig. 2a) and amine (Fig. 2b). Strong peak at 1144 cm^−1^ represents the S–N stretching (the weak band observed at the same frequency in the sevelamer spectrum) was notable. In Fig. 2a, the absorption range at 1176–1284 and 1006–1088 cm^−1^ shows the possibility of O–S–O asymmetric and symmetric stretching modes, respectively and the S\O stretching mode lies at 675–852 cm^−1^ showing the presence of the sulfonic acid functional group, which was consistent with the reported IR spectra for SO_3_H.

#### Thermogravimetric analysis

**Fig. 3:**
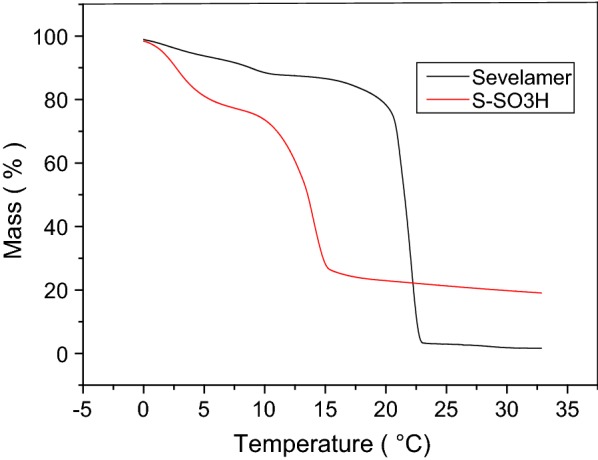
TGA of resin sevelamer and resin S-SO_3_H

The thermogravimetric analysis (TGA) of S-SO_3_H in comparison with sevelamer is shown in Fig. 3. Weight loss of (5 wt%) below 100 °C for sevelamer is displayed by the TGA curves in (Fig. 3a), which corresponds to the loss of physically adsorbed water. The ash content was only 1.6%. In the TGA curve for S-SO_3_H (Fig. 3b), three regions corresponding to different mass lose ranges exist. The first mass loss region was below 150 °C and was attributed to the loss of trapped water from the catalyst. A mass loss of approximately 10 wt% occurred between 100 and 200 °C, which was related to the slow mass loss of SO_3_H groups. Finally, a mass loss of approximately 50 wt% occurred between 240 and 300 °C, which was related to the sudden mass loss of the resin. The ash content was up to 20%. This result shows the S-SO_3_H easily turns into carbon or sulfate slats under the acidic atmosphere and high temperature. Also, from the TGA results, it was found that sevelamer has a greater thermal stability than S-SO_3_H. However, S-SO_3_H can still be safely used in organic reactions at temperatures in the range of 0–120 °C.

### BET surface area analysis

The surface area of the catalysts is an important factor influencing the catalytic activity. The surface area of polymer resin and catalyst was determined using the nitrogen gas adsorption method. BET surface area of sevelamer (2.668 m^2^ g^−1^) is almost same as the surface area of S-SO_3_H (3.010 m^2^ g^−1^).

### Application of S-SO_3_H as an efficient catalyst in organic reactions

The studies demonstrated for the use inexpensive and environmentally friendly S-supported sulfonic acid (S-SO_3_H) as a heterogeneous solid acid catalyst shows very satisfactory results. In the present work, S-SO_3_H was easily prepared and used as a highly efficient, heterogeneous, reusable and inexpensive solid acid catalyst for synthesis of 1,8-dioxo-octahydroxanthene derivatives.

The 1,8-dioxo-octahydroxanthenes are an important structural unit in many natural compounds with biological and pharmaceutical activities. Water is considered to be an environmentally benign solvent. Therefore, the reaction of benzylaldehyde and 1,3-cyclohexanedione was selected as a model reaction to screen the experimental conditions in aqueous media. Reaction progress was monitored by thin-layer chromatography. To examine the temperature effects, the model reaction was carried out with catalyst loadings of 0.010 g S-SO_3_H in aqueous media. The reaction proceeded smoothly at room temperature, but the product was obtained in 73% in 120 min (Table 1, entry 1). With an increase in temperature, the reaction time was significantly decreased. This reaction was completed in 30 min at 90 °C (Table 1, entry 4). To justify the efficiency of the S-SO_3_H, the model reaction was carried out in the presence of different amounts of S-SO_3_H in aqueous media. It was found that the product **3a** was formed in high yields with catalyst loadings of 0.0100 g (Table 1, entry 8). A higher amount of catalyst did not accelerate the reaction time or promote the reaction yield (Table 1, entry 9). A lower yield of product **3a** was obtained with a lower amount of catalyst and in a reaction time of 8 h (Table 1, entry 5).

**Table 1:** Temperature and catalyst amount for the synthesis of 1,8-dioxo-octahydroxanthene


Entry	T (°C)	Amount of catalyst (g)	Time (min)	Yield (%)^a^
1	25	0.0100	120	73
2	50	0.0100	120	82
3	70	0.0100	90	91
4	90	0.0100	30	94
5	90	–	12 h	31
6	90	0.0010	8 h	84
7	90	0.0050	60	90
8	90	0.0100	30	94
9	90	0.0150	30	95

Reaction conditions: **1a** (1.0 mmol), **2a** (2.0 mmol), S-SO_3_H (10 mg), water (1.0 mL), 90 °C

^a^Isolated yield

After optimizing the conditions, the scope of the method was successfully studied using a variety of aldehydes in water. Both electron-rich and electron-deficient aromatic aldehydes worked well, giving high yields of the desired products. Electron-deficient aldehydes needed shorter reaction times and gave somewhat higher yields than their electron-rich counterparts (Table 2, entries 1–6). In almost all cases, the reactions proceeded smoothly within 30-50 min.

**Table 2:** Substrate scope of reaction between various aromatic aldehydes and cyclohexanedione


Entry	R(Ar)–	Time (min)	Product	Yield (%)^a^	mp (°C)
1	Ph	30	**3a**	94	213–215
2	4-CH_3_Ph	45	**3b**	91	244–246
3	4-CH_3_OPh	45	**3c**	92	190–191
4	4-ClPh	30	**3d**	93	289–291
5	2-ClPh	30	**3e**	91	250–252
6	4-FPh	30	**3f**	93	277–278
7	4-NO_2_Ph	30	**3g**	90	234–236
8	Ph	50	**4a**	90	202–204
9	4-CH_3_Ph	50	**4b**	89	215–217
10	4-ClPh	45	**4c**	91	230–232
11	4-FPh	45	**4d**	90	223–225
12	2,4-diClPh	45	**4e**	88	219–221

Reaction conditions: **1** (1.0 mmol), **2** (2.0 mmol), S-SO_3_H (10 mg), water (1.0 mL), 90 °C

^a^Isolated yield

Five consecutive syntheses of 1,8-dioxo-octahydroxanthene was done to demonstrate the easy recyclability using the recovered **S-SO**_**3**_**H** catalyst. Once product **3a** was filtered from the reaction mixture, the **S-SO**_**3**_**H** catalyst was used directly in another cycle without drying. The catalyst showed no appreciable loss in activity. All reactions were completed in 30 min and the desired product was obtained in 91–94% yield.

## Conclusion

Sevelamer (polyallyamine resin)-supported sulfonic acid (S-SO_3_H) was used as an efficient heterogeneous catalyst for the synthesis of 1,8-dioxo-octahydroxanthene derivatives in summary. This catalyst could be easily separated from the reaction mixture and directly reused after a simple extraction step. The catalyst was recycled for seven consecutive cycles without any obvious loss in its catalytic activity. As a good heterogeneous solid acid, S-SO_3_H is stable in air, easy to prepare and can be recovered easily. The scope and synthetic applications of the S-SO_3_H catalyst are currently under investigation in our laboratory.

## Data Availability

All data are fully available without restriction.
